# Controlling the Adsorption and Release of Ocular Drugs in Metal–Organic Frameworks: Effect of Polar Functional Groups

**DOI:** 10.1021/acs.inorgchem.2c02539

**Published:** 2022-11-15

**Authors:** J. Farrando-Pérez, G. Martinez-Navarrete, J. Gandara-Loe, S. Reljic, A. Garcia-Ripoll, E. Fernandez, J. Silvestre-Albero

**Affiliations:** † Laboratorio de Materiales Avanzados, Departamento de Química Inorgánica-Instituto Universitario de Materiales, Universidad de Alicante, E-03690 San Vicente del Raspeig, Spain; ‡ Neuroprosthesis and Neuroengineering Research Group, Institute of Bioengineering, Miguel Hernández University, E-03202 Elche, Spain

## Abstract

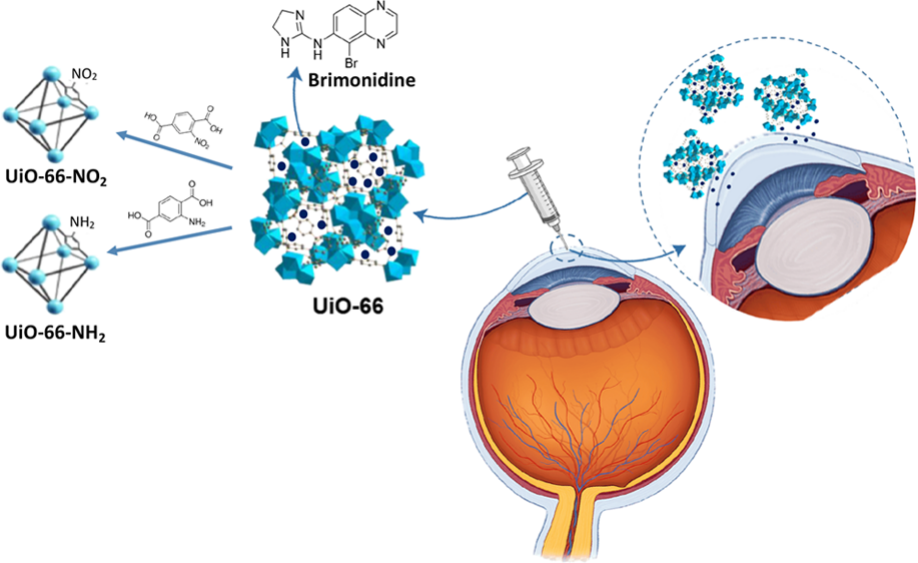

A series of UiO-66 materials with different functional groups (−H, −NH_2_, and −NO_2_) have been evaluated for the adsorption and release of a common ocular drug such as brimonidine tartrate. UiO-66 samples were synthesized under solvothermal conditions and activated by solvent exchange with ethanol. Experimental results suggest that the incorporation of surface functionalities gives rise to the development of structural defects (missing linker defects) but without altering the basic topology of the UiO-66 framework. These defects improve the adsorption performance of the parent metal–organic framework (MOF), while the bulkier functionalities infer slower release kinetics, with the associated benefits for prolonged delivery of brimonidine. Among the evaluated MOFs, defective UiO-66-NO_2_ can be proposed as the most promising candidate due to the combination of a larger brimonidine volumetric uptake (680 mg/cm^3^), a prolonged delivery (period of up to 25 days), a small particle size, and a larger instability. Contrariwise, at high concentrations UiO-66-NO_2_ has higher toxicity toward human retinal pigment epithelium cells (ARPE-19) compared to the pure and NH_2_-functionalized UiO-66.

## Abstract 2

UiO-66 derivatives are promising vehicular platforms for controlled delivery of ocular drugs. The presence of bulkier functional groups and the associated structural defects define the total uptake and the delivery kinetics.

## 1 Introduction

Metal–organic frameworks (MOFs) have been targeted as prominent porous materials in different applications such as catalysis, adsorption, sensors, drug delivery, and separation due to their extraordinary properties in terms of porosity, surface/volume ratio, chemical and thermal stability, etc.^1^ Most importantly, the advantage of MOFs over other porous materials lies in the vast number of combinations between linkers and metal centers and the ability to form and control nanosized particles, which allow the tailoring of their chemical and physical properties.^2,3^

MOFs have also been targeted as potential nanocarriers in biomedical applications due to the above-mentioned characteristics. For instance, MOFs have been reported to successfully deliver small therapeutic molecules such as anti-inflammatories^4,5^ and antibiotics^6^ and larger molecules such as proteins,^7^ nucleic acids,^8,9^ viruses,^10^ and cells.^11^ However, the introduction of chemical functionalities in the surface of the MOF material (e.g., functionalization of the organic linker) has been anticipated as an essential step in order to obtain materials tailored for targeted applications.^12,13^ For instance, Orellana-Tavra et al. reported the tuning of Zr-based MOFs through linker functionalization to control the endocytosis mechanism of cells to design efficient drug delivery systems through the evaluation of calcein.^14^ In a similar approach, Haddad et al. evaluated the functionalization of Zr-based MOFs to enhance the targeted delivery of the anticancer agent dichloroacetate (DCA) to the mitochondria, the synergic effect between the drug and the MOF structure being responsible for the improved efficacy of the anticancer agent.^15^ The adsorption of caffeine in a series of UiO-66 (UiO = Universitet I Oslo) materials with −NH_2_ and −OH functional groups was evaluated using DFT calculations, the modeling predicting a preferential adsorption of the drug in the vicinity of the organic linker and a competitive adsorption effect with water molecules in the functional groups.^16^

Another field where drug delivery nanodevices are urgently needed is ophthalmology. A critical aspect in the field of ophthalmology that makes the design of nanodevices challenging is the small size of the ocular cavity. Among the different ocular disorders that require prolonged drug exposure, open-angle glaucoma is one of the most relevant diseases. Glaucoma is a progressive eye disease that can lead to irreversible blindness, and it is the second leading cause of permanent visual deterioration worldwide.^17^ Glaucoma encompasses different diseases, all of them ending in the damage of the optical nerves, loss of retinal ganglion cells (RGCs), and elevated intraocular pressure (IOP).^18^ Statistics estimate that more than 64.3 million people are currently affected by these diseases, and it is expected that 112 million people will be suffering from glaucoma by 2040.^19^ Alpha-adrenergic drugs such as brimonidine tartrate have been essential to revert and minimize the effect of glaucoma due to their capacity to lower IOP.^20^ However, brimonidine is commonly administrated either by eye droplets, which involves the drainage of almost 97% of the active molecules by tear fluid and blinking effect, or through unpleasant intraocular injections.^21^ The periodic administration of these drugs ends up with increased side effects and poor compliance of the patients. A promising approach to solve these problems is based on the development of drug nanocarriers, i.e., nanovesicles,^22^ chitosan nanoparticles,^23^ or MOFs,^24^ to control the transport and release of brimonidine inside the ocular cavity. Unfortunately, formulations based on macromolecules or polymers suffer generally either from a low loading capacity (gravimetric <1 wt % and/or volumetric <0.1% w/v) or from fast release of the drug (within minutes/hours). Due to the restricted space of the globe, a proper candidate must combine an optimum storage capacity (on a volumetric basis), a proper biocompatibility, a prolonged delivery, and proper degradation and elimination upon use.

**Scheme 1:**
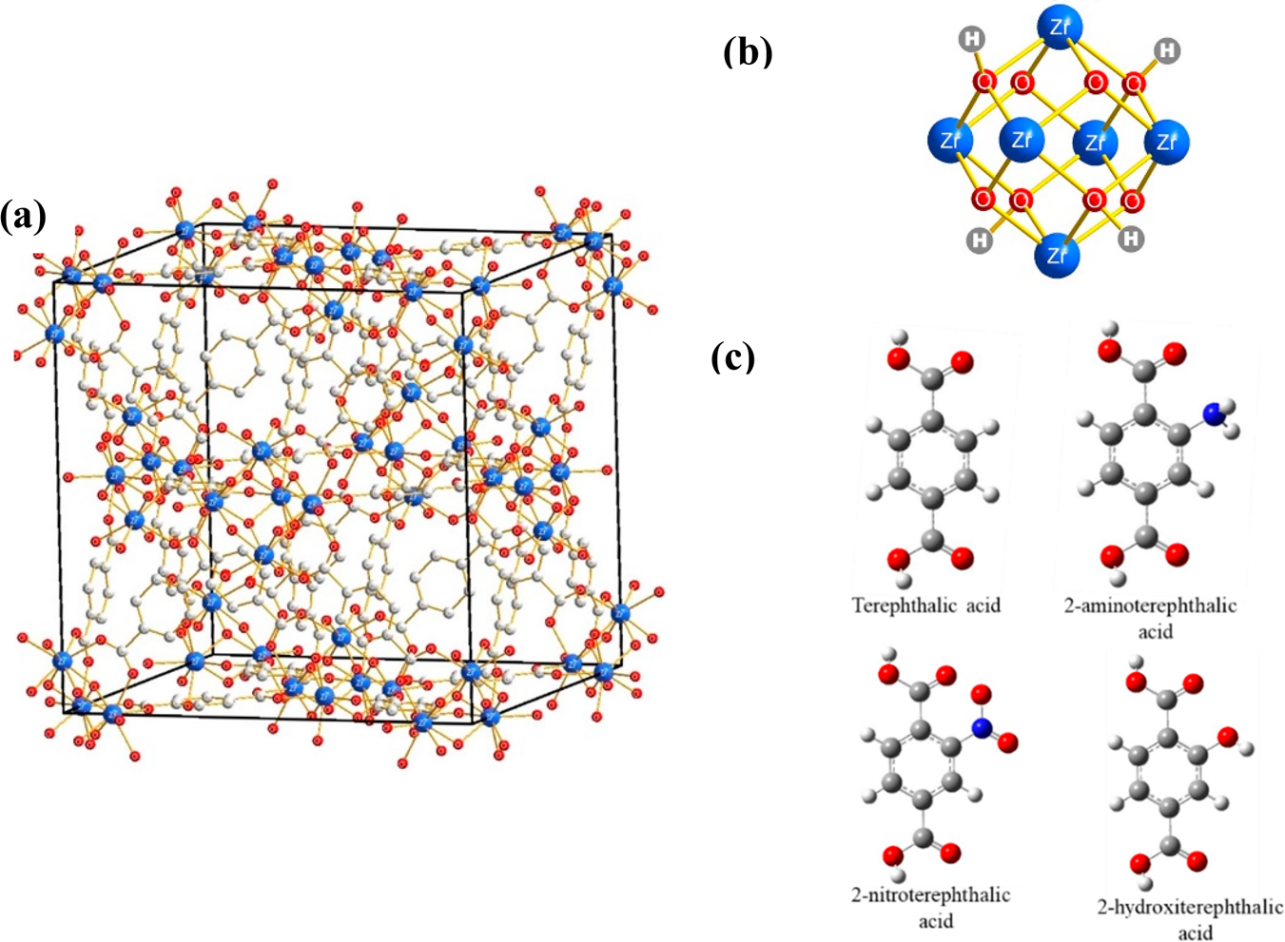
Schematic Illustration of (a) UiO-66 (Zr) Structure,^31^ (b) Zr Cluster,^31^ and (c) Terephthalic Acid (Benzene-1,4-dicarboxylic acid) Derivatives

Among the different candidates, MOF materials, and more specifically Zr-based frameworks, have been proposed in the literature as efficient nanocarriers in the adsorption and controlled release of ocular drugs (e.g., brimonidine), with a promising storage capacity, a slow release, and an associated low cytotoxicity for retinal cells.^25^ For instance, a Zr-cluster-based MOF such as UiO-66 shows a half-maximal inhibitory concentration (IC_50_) of 1.50 ± 0.15 mg/mL after 24 h of exposure.^26^ Recently, it has been reported that the adsorption capacity of UiO-66 for brimonidine is extremely high, up to 249 mg/g, and the value reaches up to 630 mg/g for a similar Zr-based MOF such as UiO-67.^25^ In both cases, the release kinetics scales up to 10–12 days. UiO-66 (Scheme 1a) is a MOF formed by the assembly of Zr clusters (Scheme 1b) and terephthalic acid as a linker. The terephthalic acid (Scheme 1c) can be easily functionalized with −NH_2_, −NO_2_, and −OH groups, thus giving rise to a wide range of new MOFs with modified physicochemical properties. Indeed, previous studies described in the literature have shown that the functionalization of the organic linker in UiO-66 gives rise to improved catalytic systems (e.g., conversion of levulinic acid), the improvement being associated with the formation of structural defects (preferentially missing linker defects).^27−29^ UiO-66 materials have also been engineered in composite materials (e.g., MOF@PU polymeric matrices) with promising results for the potential fabrication of novel ocular devices (i.e., contact lenses and punctual plugs) for prolonged release of ocular drugs.^30^

Based on these premises, the aim of this work is to evaluate the effect of the linker functional group (−H, −NO_2_, −NH_2_) and, indirectly, the effect of the structural defects, in the uptake and controlled release of brimonidine in physiological media and using UiO-66 as a guest structure. The host–guest interactions upon adsorption will be carefully analyzed to ascertain the role of the functionalities in the adsorption/desorption process. The main goal will be to extend the release kinetics so that prolonged exposure devices can be designed without compromising the excellent storage capacity of the parent MOF.

## 2 Experimental Section

### 2.1 Synthesis of MOFs

UiO-66 and its derivatives had been synthesized following the procedure described by Katz et al.^32^ For UiO-66, 0.5 g of ZrCl_4_ was dissolved in 20 mL of dimethylformamide (DMF) and 4 mL of concentrated HCl. In a second vessel, 0.492 g of terephthalic acid (BDC) was dissolved in 40 mL of DMF. The two solutions were mixed and continuously stirred for 30 min. The colorless solution was transferred to a 200 mL jar which was tightly closed and kept at 80 °C overnight. The resulting solid was filtered and washed first with DMF (2 × 30 mL) and then with ethanol (2 × 30 mL). The sample was activated using an outgassing treatment at 150 °C for 3 h under ultrahigh vacuum conditions. The same procedure was used to synthesize the UiO-66-NO_2_ and UiO-66-NH_2_ samples, but replacing BDC by 0.6282 g of 2-nitrotherephthalic acid (BDC-NO_2_) and 0.5394 go of 2-aminotherephthalic acid (BDC-NH_2_), respectively.

### 2.2 MOF Physicochemical Characterization

#### 2.2.1 X-ray Diffraction (XRD) Measurements

The crystallographic quality of the synthesized MOFs was evaluated using X-ray diffraction (XRD). XRD patterns were recorded using a Bruker D8-ADVANCE instrument equipped with a Goebel mirror with a high-temperature chamber and an X-ray generator (KRISTALLOFLEX K 760-80F) with an XR tube with a copper anode. XRD patterns were recorded between 3° and 60° (2θ) with a step of 0.05° and a time per step of 3 s.

#### 2.2.2 Nitrogen Isotherms at −196 °C

The textural characteristics of the synthesized MOFs were evaluated by nitrogen adsorption–desorption isotherms at −196 °C. Gas physisorption measurements were performed in a homemade fully automated manometric instrument designed and developed by the Advanced Materials Laboratory (LMA). Prior to the adsorption measurements, the samples were outgassed under ultrahigh vacuum conditions at 150 °C for 8 h. Specific surface area was obtained after application of the BET equation, and total pore volume was determined from the amount adsorbed at *p*/*p*_0_ = 0.97. Micropore volume (*V*_micro_) was obtained using the Dubinin–Radushkevich (DR) model equation with the nitrogen adsorption data. Finally, mesopore volume (*V*_meso_) was obtained from the difference *V*_total_ – *V*_micro_.

#### 2.2.3 Thermogravimetric Analysis (TGA)

The thermogravimetric experiments were carried out in a Mettler Toledo TGA/SDTA instrument. To obtain the thermograms, an alumina crucible containing 10 mg of sample was heated under 100 mL min^–1^ of N_2_ flow up to 1000 °C, at a heating rate of 10 °C min^–1^.

#### 2.2.4 Scanning Electron Microscopy (SEM)

The morphology of the synthesized MOFs was evaluated using field-emission scanning electron microscopy (FESEM). These analyses were performed in a Merlin VP Compact system from ZEISS with a resolution of 0.8 nm at 15 kV and 1.6 nm at 1 kV.

#### 2.2.5 Fourier Transform Infrared Spectroscopy (FTIR) Measurements

Fourier transform infrared spectroscopy measurements were recorded in a JASCO FTIR 4700 spectrometer with a resolution of 2 cm^–1^, a Germanium encapsulated KBr beam splitter, and a DLaTGS detector.

#### 2.2.6 X-ray Photoelectron Spectroscopy (XPS) Measurements

XPS analyses were performed in a K-ALPHA Thermo Scientific instrument. XPS spectra were collected using a Al K radiation (1486.6 eV), monochromatized by a twin crystal monochromator, yielding a focused X-ray spot elliptical shaped with a major axis length of 400 mm at 3 mA × 12 kV. The alpha hemispherical analyzer was operated in the constant energy mode with survey scan pass energies of 200 eV to measure the whole energy band and 50 eV in a narrow scan to selectively measure the desired elements. Charge compensation was achieved with the system flood gun that provides low-energy electrons and low-energy argon ions from a single source. The CH_*x*_ in the carbon 1s score level was used as the reference binding energy (284.6 eV). The powder samples were pressed and mounted on the sample holder and placed in the vacuum chamber. Before the spectrum recording, the samples were maintained in the analysis chamber until a residual pressure of ca. 5 × 10^–7^ N m^–2^.

### 2.3 Brimonidine Loading and Release Experiments

The amount of the brimonidine tartrate adsorbed in the different samples was evaluated using high-performance liquid chromatography, following the method developed by Karamanos et al.^33^ Briefly, a 1500 ppm stock solution was prepared dissolving 1.5 g of brimonidine tartrate in 1000 mL of ultrapure water. The calibration curve was performed measuring concentrations from 2 to 15 ppm using a 1260 Infinity Binary LC chromatograph (Agilent Tech.), equipped with an Agilent Lichrospher 100RP-18 (5 μm, 4 × 250 mm) column. The mobile phase was 10% (v/v) acetonitrile in 10 mM triethylamine buffer, pH 3.2, and ACN (90:10). The separation was performed at room temperature, at a flow-rate of 1.0 mL/min, with an injection volume of 5 μL, with the detection of brimonidine at 248 nm, using a DAD (diode array detector). This method gave an accuracy above 97%, thus confirming the validity of the HPLC technique for brimonidine determination.

For the loading tests, aqueous solutions with initial concentrations of 250, 500, 750, 1000, and 1500 ppm brimonidine tartrate were prepared from the original stock solution. 100 mg portions of each MOF (previously outgassed at 150 °C overnight) were placed in contact with 50 mL of each solution and left under stirring until equilibrium was reached. The quantification of brimonidine tartrate was determined by diluting aliquots at ratios of 1:100 and using the method described above. The kinetic behavior of each MOF was evaluated by taking aliquots at different time intervals.

For the release tests, 100 mg of MOF was first introduced into 50 mL of a 1500 ppm brimonidine tartrate aqueous solution. Once the sample reached equilibrium, it was collected by filtration, and an aliquot was saved to determine the maximum loading capacity. The brimonidine-loaded MOF was washed several times with ultrapure water and dried under a vacuum at 60 °C for 6 h. In order to carry out the brimonidine release, dried samples were placed in 50 mL of physiological solution (PBS). Brimonidine determination with time was performed using the same procedure described above, using PBS as a solvent instead of ultrapure water.

### 2.4 Cell Culture and MTT Assay

The ARPE-19 cell line from human retinal pigment epithelium was purchased from the American Type Culture Collection (ATCC, Manassas, VA). Cells were plated at a density of 10 000 cells/well in a 96-multiwell plate and cultured in Dulbecco’s modified Eagle’s medium mixed with Ham’s F-12 medium (Thermo Fisher Scientific) with 10% fetal bovine serum (Thermo Fisher Scientific) and 1% penicillin–streptomycin (Thermo Fisher Scientific).

Cells were treated with MOFs at concentrations ranging from 0.1 to 10 mg/mL. After 24 h, cell viability was analyzed using a 3-(4,5-dimethylthiazol-2-yl)-2,5-diphenyltetrazolium bromide (MTT) assay. Briefly, media with MOFs were aspirated, and cells were incubated with 0.05 mg/mL MTT reagent (Sigma-Aldrich) for 4 h at 37 °C. MTT was removed, and the formazan crystals were dissolved in dimethyl sulfoxide (DMSO). The absorbance was read at 595 nm using an iMark microplate reader. The cell viability was represented as a relative percentage with respect to the untreated control (positive control). Cells treated with ethanol for 15 min were taken as a death control (negative control).

## 3 Results and Discussion

### 3.1 Physicochemical Characterization of the Synthesized MOFs

**Figure 1:**
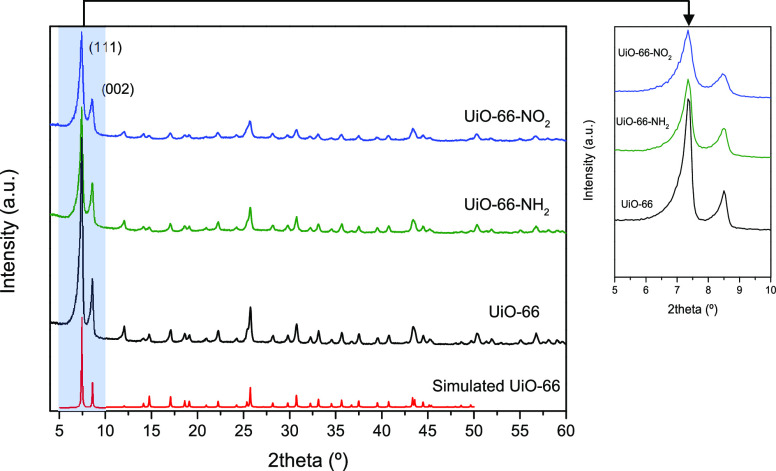
XRD patterns of the UiO-66 sample and the functionalized (−NO_2_ and −NH_2_) equivalents. The simulated pattern for pure UiO-66 has been included for a comparison.

The crystallinity of the three synthesized MOFs was evaluated using XRD measurements. Figure 1 shows the XRD patterns for the UiO-66 and the derivatives evaluated within a 2θ range of 3–50°. The XRD patterns perfectly fit with those previously described in the literature for these materials, thus confirming the quality and reproducibility of the three MOFs synthesized by the solvothermal method.^32^ These results confirm the successful growth of the functionalized UiO-66 topological equivalents with two main peaks at ca. 7.4° and 8.5°, corresponding to (111) and (002) planes of the UiO-66 crystal series, respectively.^34^ At this point, it is interested to highlight that, in spite of the proper crystallinity, functionalization gives rise to significant changes in the *I*_(111)_/*I*_(002)_ ratio. Previous results described in the literature have shown that these variations are a clear indication of the presence of structural defects in UiO-66 samples.^32,35^ More specifically, these changes are consistent with the presence of missing linker defects (the 002 reflection is systematically more intense when linkers are missing). The *I*_(111)_/*I*_(002)_ ratio changes from 2.8 in the nonfunctionalized UiO-66 down to 1.4 for UiO-66-NH_2_ and 1.2 for UiO-66-NO_2_ (see the inset in Figure 1).

**Figure 2:**
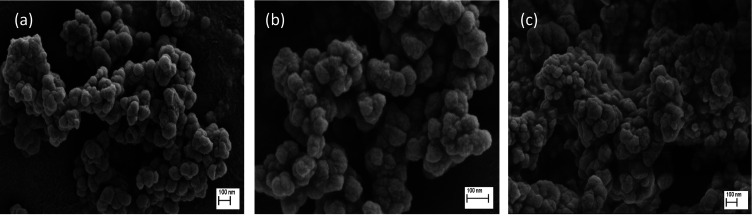
FESEM images of UiO-66 (a), UiO-66-NO_2_ (b), and UiO-66-NH_2_ (c).

The morphology and particle size of synthesized nanomaterials are essential parameters to take into consideration for their use in biomedical applications. The particle size of MOFs determines not only the biodistribution and the translocation in the human body but also, most importantly, the degradation and excretion from the human body after use.^36^ Therefore, it is extremely important to obtain small crystals, preferentially when dealing with ocular disorders, to minimize any potential limitation in the visual field. The shape and size of the synthesized materials have been evaluated using FESEM. Figure 2 shows the FESEM images of UiO-66 and the functionalized derivatives. As can be observed, all three materials show particle aggregates in the nanoscale range. More specifically, in the case of UiO-66 and the derivative UiO-66-NO_2_, a homogeneous particle size distribution is observed with an average particle size ca. 80 ± 10 nm. Contrariwise, the UiO-66-NH_2_ sample is more heterogeneous with particles in the size range 40–150 nm.

**Figure 3:**
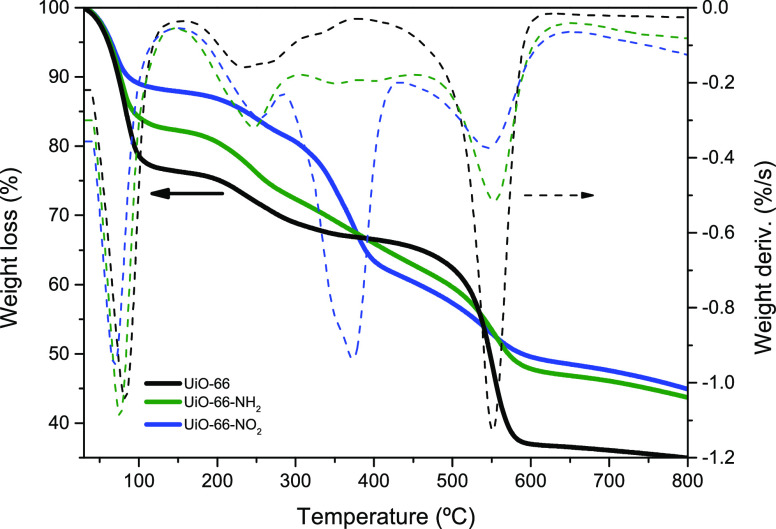
TGA and DTGA analysis of UiO-66 samples and the functionalized derivatives.

The thermal performance of the synthesized MOFs (stability and integrity of the UiO-66) and its derivatives was evaluated using thermogravimetric analysis (TG-DTGA). Figure 3 shows the thermogravimetric profile in the temperature range 25–800 °C, using air as the gas carrier. All three materials exhibit a similar thermogravimetric profile with three well-defined weight loss steps at low (∼80 °C), medium (∼250–400 °C), and high (>500 °C) temperatures.^27^

The first step close to 100 °C is attributed to the loss of the free solvent (ethanol) that remains in the cavities of the synthesized materials and moisture. In addition, all samples exhibit a small weight loss at ca. 250 °C, more intense for the functionalized samples, probably attributed to the desorption of DMF remaining in the UiO-66 cavities after the solvent exchange process or the removal of uncoordinated linkers. Above this temperature, UiO-66 and UiO-66-NH_2_ samples exhibit a significant thermal stability up to 550 °C. At this temperature, both samples suffer a severe deterioration due to the decomposition of the organic linker and degradation of the MOF structure.^30^ In the specific case of the more polar −NO_2_ functionality, the degradation of the structure already starts at lower temperatures with an important weight loss at 350 °C, most probably promoted by the presence of structural defects.^37^ At this point, it is interesting to highlight that whereas the nonfunctionalized UiO-66 loses more than 60% of its weight at 800 °C, functionalized derivatives lose less than 55% of the original weight. The higher residual weight at 800 °C in the functionalized MOFs could be another indication of the structural defects (missing linkers) anticipated by the XRD measurements.^32,35,38^ From the TG analysis, a higher number of defects can be inferred for samples UiO-66-NH_2_ (after removing humidity), although UiO-66-NO_2_ has a larger structural instability.

**Figure 4:**
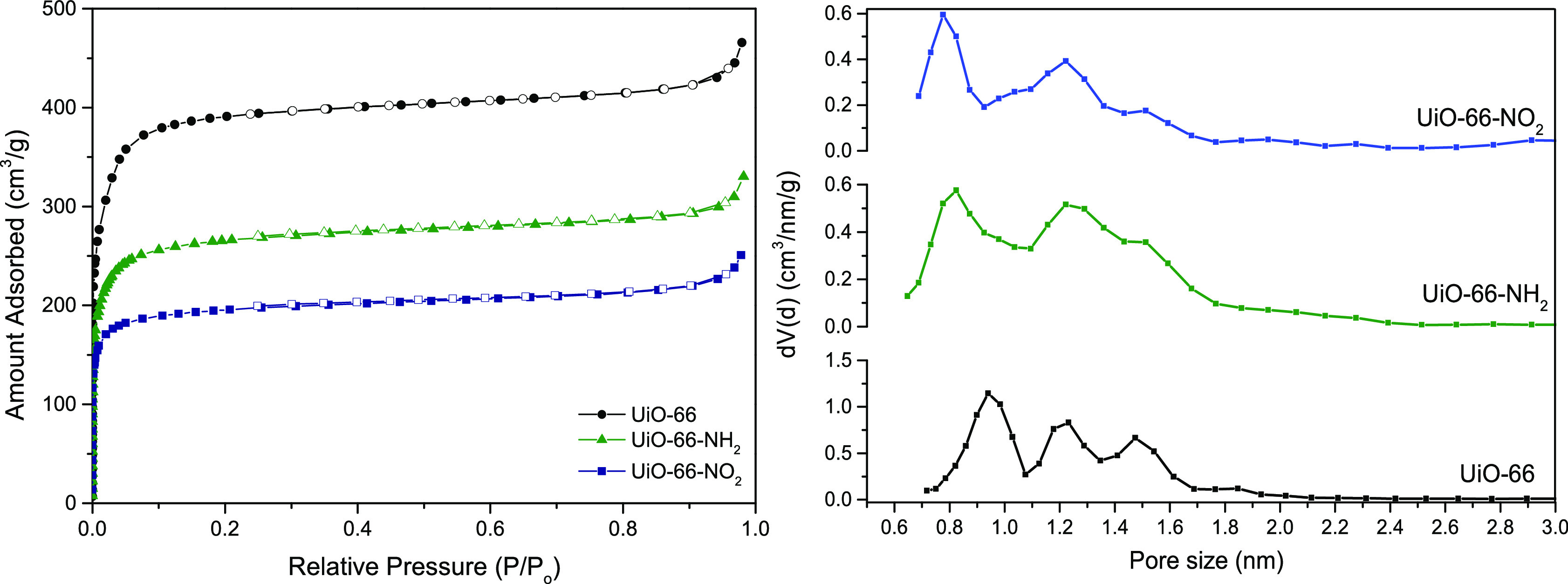
(left) Adsorption–desorption isotherms at −196 °C in UiO-66 and its derivatives. (right) Pore size distribution obtained after application of the NLDFT model.

The textural properties of the synthesized materials were evaluated using nitrogen adsorption at −196 °C. Figure 4 shows the N_2_ adsorption–desorption isotherms of the synthesized MOFs. All three samples exhibit a type I isotherm, according to the IUPAC, characteristic of microporous solids. The original UiO-66 has a BET surface area up to 1455 m^2^/g, which is slightly larger than the values reported in the literature.^32,39^ This high value (quite above the theoretical prediction of 950 m^2^/g) can be explained due to the absence of a small fraction of linkers (structural defects). Katz et al. reported that acid addition to the synthesis media of UiO-66 is a very useful method to optimize the surface area of the material through the selective elimination of 4 linkers per node and, as a consequence, the associated increase in the microporosity of the material.^32^ Nevertheless, the polar −NH_2_ and −NO_2_ derivatives present a reduction in the surface area of ca. 30% and 50%, respectively (Table 1). This decrease in surface areas could be explained due to the insertion of relatively heavy and bulkier polar functional groups −NH_2_ and −NO_2_ in the 3D network of the material.^32,40,41^ The pore size distribution (PSD) for the three evaluated materials is rather similar with a main contribution at ca. 0.9 nm and two broad peaks in the large micropore range (above 1 nm), due to the presence of different pores (octahedral pores) in UiO-66 (defective structure due to the use of HCl in the synthesis). However, a closer look at Figure 4 shows that, upon functionalization, the main contribution (due to small tetrahedral pores) is slightly shifted to lower values (ca. 0.8 nm), while contributions above 1 nm become broader and less defined. These observations would be *a priori* in agreement with a more heterogeneous porous structure upon functionalization, most probably associated with a more defective network (e.g., increasing average pore size of the large octahedral pores and development of narrow micropores).^32^

**Table 1:** Textural Properties of the Synthesized MOFs^a^

sample	SBET (m^2^/g)	*V*_o_ (cm^3^/g)	*V*_t_^b^ (cm^3^/g)
UiO-66	1455	0.53	0.69
UiO-66-NH_2_	1011	0.37	0.51
UiO-66-NO_2_	740	0.31	0.39

a Micropore volume (*V*_0_) obtained after application of the Dubinin–Radushkevich equation.

b Total pore volume calculated at *P*/*P*_o_ = 0.90.

As a result of the insertion of the polar functional groups in the linker, textural parameters also reflect a decrease (ca. 30–40%) in the micropore volume and total pore volume (Table 1) for the modified samples compared to the original UiO-66.^41^ This observation agrees with the decrease observed above in the BET surface area upon functionalization.

**Figure 5:**
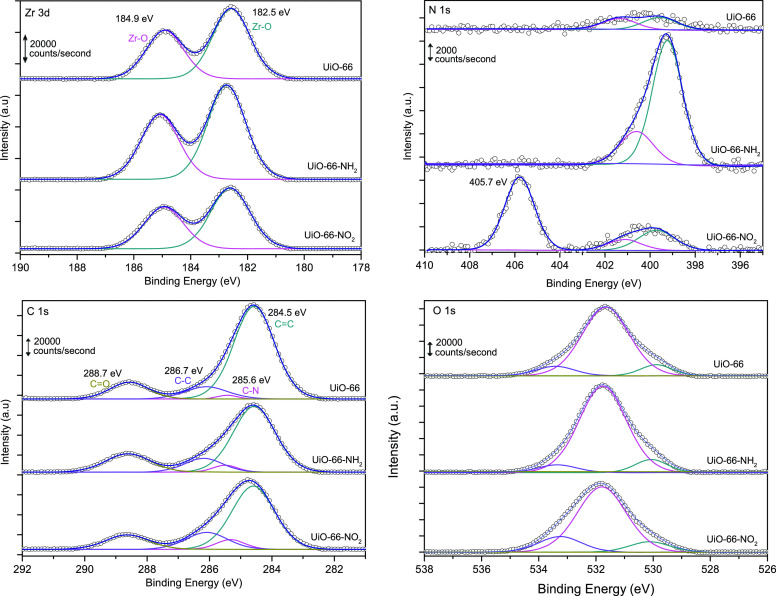
XP spectra in the Zr 3d, N 1s, C 1s, and O 1s regions for the three samples evaluated.

The chemical composition of the MOF framework has been evaluated using X-ray photoelectron spectroscopy (XPS). Figure 5 shows the XPS analysis for the three MOFs evaluated in the Zr 3d, N 1s, and O 1s regions. For all three samples, the Zr 3d spectrum exhibits two well-defined contributions at 184.9 and 182.5 eV, corresponding to the Zr 3d_5/2_ and 3d_3/2_ contributions, respectively. These contributions are associated with the presence of Zr—O and Zr—Zr environments in the three MOFs evaluated.^42^ At this point, it is important to highlight that both Zr contributions exhibit a slight shift to higher binding energies (185.1 and 182.7 eV) upon functionalization with —NH_2_. This shift reflects the electron deficiency state of the Zr—O clusters due to the high concentration of missing linker defects.^38^ The N 1s spectrum shows different contributions depending on the functionalization performed. In the original UiO-66-H, the signal is rather small due to the absence of nitrogen in the MOF network. In the —NH_2_-functionalized material, two well-defined contributions can be appreciated at 399.2 and 400.6 eV, associated with C—N species (amine).^42^ In the case of the UiO-66-NO_2_, the C—N contributions at 399.6 and 401.1 eV are small (similar to the original UiO-66), and a new intense band appears at 405.7 eV attributed to —NO_2_ groups linked to an aromatic ring.^43^ The C 1s spectra show several contributions at 284.5, 285.6, 286.7, and 288.7 eV, attributed to C=C, C—N, C—C, and C=O, respectively. As expected, these contributions are rather similar for the three samples evaluated, except the C—N signal that is smaller in the original UiO-66-H sample. Finally, the O 1s spectra are rather similar for the three MOFs evaluated with three well-defined contributions, a main one at 531.6 eV and two shoulders at 529.8 and 533.4 eV. These contributions correspond to —OH, Zr—O, and O—C=O, respectively.^38^ Similarly to Zr 3d, the main oxygen peak in the functionalized samples is shifted to higher binding energies (531.8 eV), thus reflecting the presence of defects.

XPS measurements are also very useful to quantify the surface amount of the different species present in the synthesized material. Table 2 summarizes the atomic percentage for the different species analyzed with XPS in the three samples evaluated. XPS data confirm the presence of C, N, O, and Zr in the composition of the evaluated MOFs. As expected, the amount of nitrogen is residual in the original UiO-66-H, and it increases significantly upon functionalization with the N-based polar groups. In addition, the Zr/C ratio has been estimated as a measure to identify potential structural defects. In close agreement with XRD, XPS, and TGA data, the amount of Zr increases for the functionalized UiO-66 samples, most probably due to the presence of missing linker defects (UiO-66-NH_2_ > UiO-66-NO_2_).

**Table 2:** Atomic Percentage for the Different Species in Three Samples

sample	%C	%N	%O	%Zr	Zr/C
UiO-66	65.7	0.7	29.3	4.3	0.065
UiO-66-NH_2_	56.9	4.5	33.3	5.2	0.091
UiO-66-NO_2_	58.8	3.5	33.1	4.6	0.078

### 3.2 Brimonidine Adsorption–Release Measurements

The brimonidine adsorption performance was evaluated using liquid-phase adsorption processes (adsorption kinetics and adsorption isotherms) in the UiO-66 and its derivatives. The isotherms were measured at 25 °C using a concentration range from 250 to 1500 ppm. Experiments were performed using ultrapure water as a solvent and 0.1 g of MOF.

**Figure 6:**
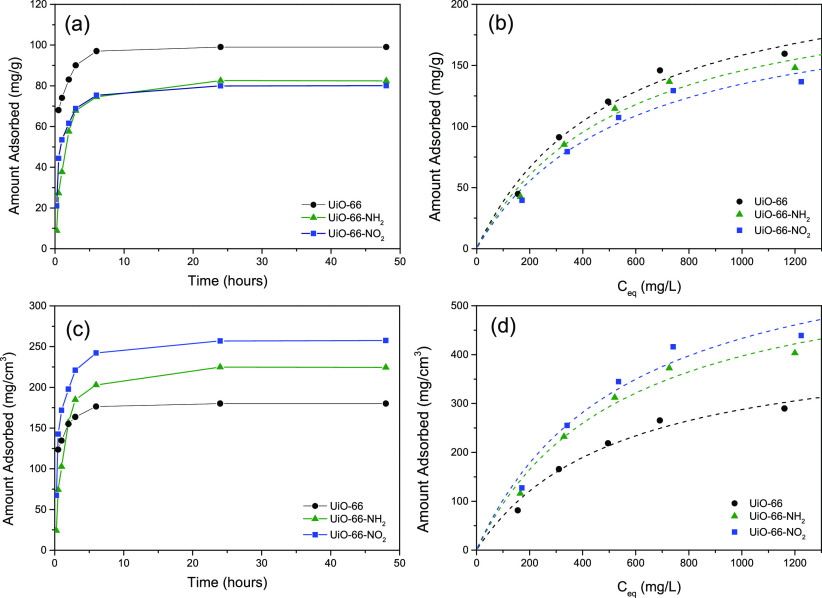
Brimonidine adsorption kinetics (a, c) and adsorption isotherms (b, d) at 25 °C for the original UiO-66 sample and its derivatives expressed as a function of the total amount of MOF on a gravimetric basis (mg/g) (a, b) or normalized by the micropore volume (mg/cm^3^) (c, d).

In a first step, the adsorption kinetics were measured in order to determine the optimum saturation of the sample and the amount adsorbed under equilibrium conditions. As shown in Figure 6a, the adsorption kinetics are rather similar for all three samples. In all cases, adsorption is fast in the initial stages (ca. 5–10 h), saturation being reached after 24 h of exposure to brimonidine solution. However, a closer look at Figure 6a suggests that adsorption kinetics are slightly faster for UiO-66; i.e., equilibrium time is reduced down to 5 h, followed by UiO-66-NO_2_ and finally UiO-66-NH_2_. This observation is understandable taking into account the absence of functional groups at the pore mouth of the original UiO-66; a similar equilibration time (∼5 h) was reported in the literature for brimonidine adsorption in nonfunctionalized UiO-66.^25^ The presence of the polar and bulkier functional groups at the pore mouth in UiO-66-NH_2_ and UiO-66-NO_2_ may explain the decrease observed in the adsorption kinetics. Either steric and/or thermodynamic effects may account for the observed performance.

The adsorption isotherms of the UiO-66 and its derivatives are shown in Figure 6b. All experimental isotherms exhibit a very good agreement with the Langmuir model (dashed line). Using the Langmuir equation, the maximum adsorption capacity for UiO-66 was estimated at ca. 200 mg/g. Upon functionalization, the amount adsorbed decreased slightly down to 190 and 180 mg/g for the −NH_2_- and −NO_2_-functionalized counterparts, respectively. The decrease observed in the total amount adsorbed is, *a priori*, expectable considering the decrease observed in the textural parameters deduced from the nitrogen adsorption data. Cunha et al. observed a similar decrease in caffeine loading in UiO-66 upon functionalization with −NH_2_, −OH, and −NO_2_ groups.^44^ The observed performance was attributed to the hydrophilic character and hydrogen bond acceptor capacity of the aforementioned functional groups grafted in the organic linker and their preferential interaction with the solvent (water) used to incorporate the drug. Under these conditions, the enhanced water–framework interactions limit caffeine uptake due to the preferential blocking of the smaller tetrahedral cages. However, a careful evaluation of the adsorption results described so far (Figures 4 and 6) clearly shows that the micropore volume of the UiO-66 decreases down to 44% upon functionalization (e.g., for the −NO_2_ counterpart), while the amount of brimonidine adsorbed decreases by only 10%, despite the presence of the solvent (water); i.e., brimonidine adsorption at 25 °C in the liquid phase is less sensitive to the presence of the surface functional groups compared to nitrogen adsorption at −196 °C in the gas phase. In order to gain some insight regarding the adsorption performance, the amount of brimonidine adsorbed was normalized by the micropore volume obtained from the nitrogen adsorption data. The main idea here is to compare the number of molecules adsorbed per unit volume available in the UiO-66-x (x = −H, −NH_2_, and −NO_2_) cavities for all samples evaluated. As shown in Figure 6d, the scenario changes completely after normalization (mg/cm^3^). The adsorption capacity for UiO-66 at saturation is the lowest among the evaluated MOFs, with a maximum uptake according to the Langmuir model of 460 mg/cm^3^. Interestingly, the normalized adsorption capacity increases for the functionalized samples with a maximum uptake at saturation of 610 and 680 mg/cm^3^, for UiO-66-NH_2_ and UiO-66-NO_2_, respectively. The enhancement in the amount adsorbed at saturation (close to a 50% increase) must be attributed to either (i) an enhanced packing density of brimonidine inside the UiO-66 cavities promoted by the polar functional groups grafted on the organic linker (adsorbate molecular size–adsorbent cage size effect), (ii) a promoting effect of surface defects in the adsorption of brimonidine, or (iii) the enhanced adsorption in the external surface in the functionalized MOFs. Previous studies described in the literature have anticipated a promoting effect of the polar functional groups for gas-phase adsorption processes, the enhanced gas–solid interactions being attributed to the polarity of the incorporated groups and the adsorbate molecular size–adsorbent cage size effects.^45−47^ In any case, the large uptake achieved with UiO-66-x samples confirms the excellent performance of these Zr-based MOFs for liquid-phase drug storage applications due to the combined presence of small tetrahedral (ca. 0.75 nm) and large octahedral (1.1 nm) cages ready to participate in the adsorption process. Furthermore, the enhanced volumetric storage capacity in the functionalized samples is crucial for drug delivery processes in restricted spaces, such as the globular eye.

**Figure 7:**
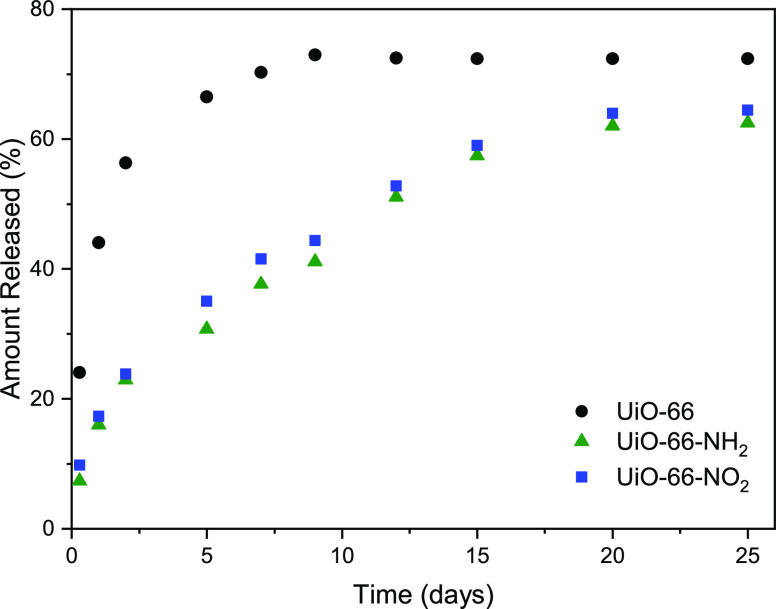
Brimonidine release performance in UiO-66 and its derivatives.

In addition to the brimonidine payload in the different Zr-based MOFs, another critical parameter to be evaluated in drug nanocarriers is the release performance. The behavior of the loaded MOFs under physiological conditions is of paramount importance to determine the long-term performance of the nanodevice and to avoid overdose symptoms in case of fast release. Previous studies from our research group using UiO-66-H for brimonidine loading and release have shown that this system can deliver a considerable amount of drug (up to 70% of its capacity) during 10 days in physiological media.^25^ Although these results are highly encouraging, more extended release periods are needed to minimize patient inconvenience. Figure 7 compares the release profiles for UiO-66 and its derivatives in physiological solution after saturation with 1500 ppm. As anticipated before, unmodified UiO-66-H exhibits a relatively fast release with more than 70% released in the first 10 days. Interestingly, the scenario changes upon functionalization. For both functional groups, release kinetics are significantly reduced with up to 25 days needed to release 60–70% of the total drug incorporated. This performance emphasizes the crucial role of the polar groups grafted on the organic linker, and the associated structural defects, in modified UiO-66 systems defining the loading and release performance for brimonidine. Release kinetics approaching 1 month are very convenient for patients to avoid undesirable daily or weekly administration protocols.^48^

Previous results described in the literature have shown that vesicular formulations and DNA nanoparticles exhibit, in general, a low loading capacity for brimonidine tartrate (ca. 17 wt % for vesicles) and a release period of only a few hours.^22,49,50^ Only chitosan inserts and poly(l-lactic acid) shells are able to surpass UiO-66 samples with a total loading capacity of ca. 38 and 48 wt %, respectively, and a release period above 30 days.^23,51^ For MOFs, UiO-67 exhibits the best performance reported so far with a total loading capacity as high as 63 wt %, highly above pure and functionalized UiO-66, although with delivery kinetics not exceeding 10–12 days.^25^

**Figure 8:**
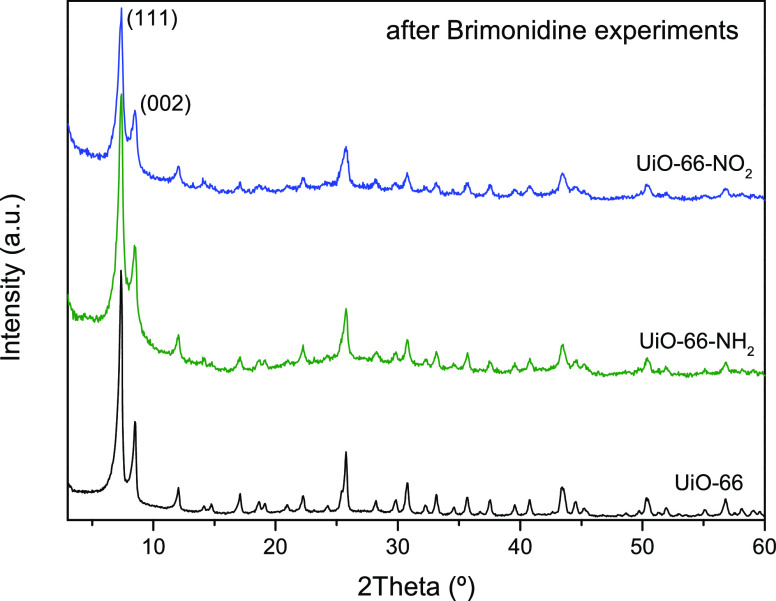
XRD patterns of the UiO-66 sample and the functionalized (−NO_2_ and −NH_2_) equivalents after brimonidine adsorption/release experiments.

Last but not least, the stability of the evaluated MOFs after the adsorption and release experiments has been evaluated using XRD analysis. Figure 8 clearly shows that the three samples retain the original crystallinity, without any sign of crystal deterioration or damage after use.

An open question at this point concerns the real location of brimonidine in the UiO-66 systems. To gain some insight in this respect, the characteristics of the loaded samples have been evaluated using thermogravimetry, X-ray diffraction, and FTIR. The main goal is to identify structural changes upon adsorption or structural details that can shed some light on the adsorption mechanism.

### 3.3 Structural Characterization of Preloaded Samples

**Figure 9:**
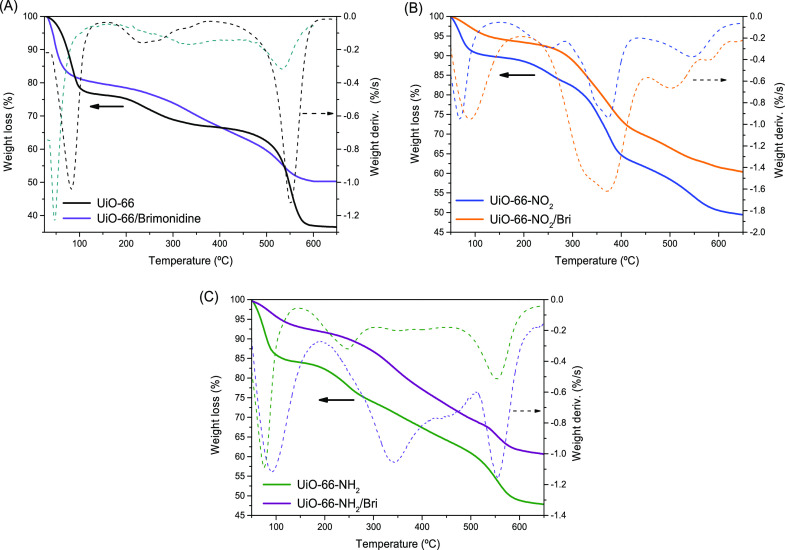
Thermogravimetric analysis (TGA and DTGA) of the different UiO-66-x samples before and after loading brimonidine.

In a first step, the characteristics of the loaded samples and their thermal stability have been evaluated via thermogravimetric analysis in an air atmosphere. Figure 9 shows the mass loss profile for the different UiO-66-x samples before and after brimonidine adsorption. As described above, original UiO-66-H exhibits three decomposition steps at low, medium, and high temperature. The first two steps at low and medium temperature (<100 and 200–300 °C) correspond to the solvent elimination, while the large weight loss at high temperatures (above 500 °C) corresponds to the decomposition of the MOF structure and the formation of ZrO_2_. The loaded sample preserves a similar TG profile below 500 °C, thus confirming that the loaded sample contains some solvent (water) occluded in the inner cavities upon adsorption. This observation is in close agreement with the slightly hydrophilic character of the UiO-66-H structure and the competing effect of water upon brimonidine loading. Interestingly, the presence of brimonidine loaded in the UiO-66-H cavities significantly inhibits the high-temperature decomposition peak. In other words, the presence of brimonidine inside the cavities enhances the structural stability of the MOF network, thus minimizing the decomposition of the organic linkers and the associated collapse of the structure. The promoting effect of adsorbed brimonidine in the structural integrity of MOFs has already been described in the literature for composite materials, UiO-67@PU films.^30^ At this point, it is interesting to highlight the absence of appreciable decomposition peaks at 210 °C (normal temperature for brimonidine decomposition),^30^ thus confirming the reciprocal effect, i.e. the promoting effect of the MOF structure in the thermal stability of the encapsulated drug. This observation suggests that brimonidine adsorption must take place preferentially inside the UiO-66 cavities, thus excluding important contributions from the external surface to the adsorption process.

A similar scenario takes place for the −NO_2_- and −NH_2_-functionalized MOFs. Figure 8 shows that the TG profiles are rather similar for the unloaded and loaded samples, except for the initial humidity that is highly reduced in the loaded samples. This observation can be explained in that the TG analysis could not be performed right after the adsorption process. Excluding this humidity effect, TG analyses confirm a synergetic effect between the occluded brimonidine molecule and the UiO-66-x network in terms of thermal and structural stability; i.e., a higher stability can be appreciated above 500 °C for the loaded materials.

**Figure 10:**
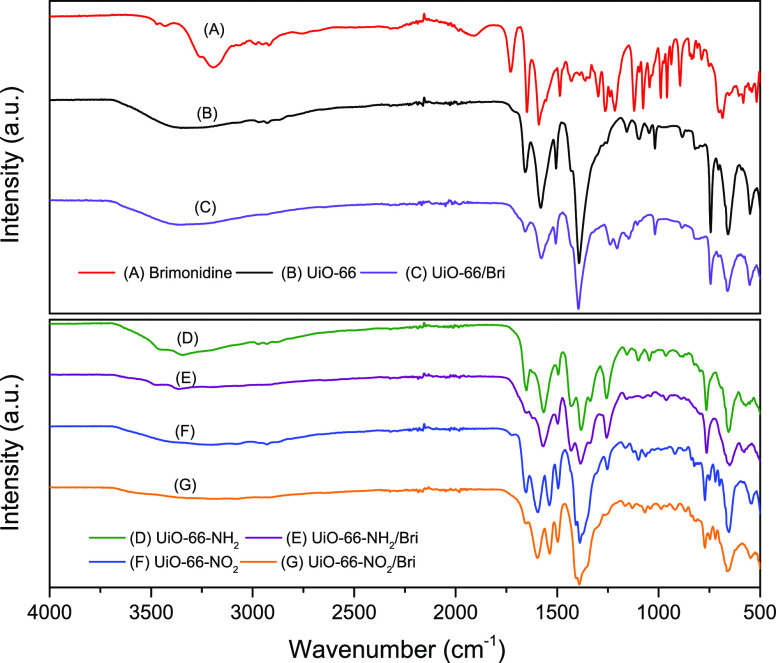
FTIR spectra of the different UiO-66-x samples before and after loading brimonidine.

Brimonidine-loaded UiO-66-x (x = —H, —NH_2_, —NO_2_) samples have also been evaluated using FTIR (Figure 10). The spectrum of the isolated drug (brimonidine tartrate) is also shown for a comparison. The original brimonidine tartrate presents characteristic vibrations at 3212 and 3268 cm^–1^ attributed to the N—H stretching vibrations from the secondary amine groups (RR′—- NH). In addition, the spectrum is dominated by strong bands at 1600–1730 cm^–1^, attributed to C=O stretching vibrations. On the other hand, the FTIR spectra of the UiO-66 samples are dominated by the vibrational modes of the organic linker with characteristic peaks in the 1382–1432 and 1565–1603 cm^–1^ ranges, corresponding to stretching vibrations (asymmetric and symmetric) from the carboxylate group of the ligands; a peak at 1537 cm^–1^, due to asymmetric N—O vibration (present for UiO-66-NO_2_); a shoulder at 1350 cm^–1^, due to the C—N stretching vibration of the aromatic amines; and peaks below 1000 cm^–1^ corresponding to Zr—O stretching vibrations.^34^ In addition, the unloaded MOFs exhibit characteristic bands at 1650–1660 cm^–1^, associated with unreacted or uncoordinated H_2_BDC linker molecules.^52^ Although this band is typical in nonactivated samples, we observe this contribution even when the samples were submitted to a thermal treatment at 150 °C for 3 h under ultrahigh vacuum conditions. Interestingly, upon adsorption, the FTIR spectra of the loaded materials remain mainly unchanged, except for the complete suppression of the peaks associated with uncoordinated H_2_BDC groups at 1650–1660 cm^–1^. A closer look at Figure 10 shows the additional suppression of the band at 1100 cm^–1^ in all samples upon loading brimonidine and the band at 1250 cm^–1^, for the —NO_2_-based UiO-66. The suppression of these FTIR bands upon adsorption provides some insight about the preferential adsorption of brimonidine molecules in the inner cavities of UiO-66-x samples and, more specifically, in the vicinity of the uncoordinated sites of the linker.

### 3.4 Cytotoxicity Studies of the Synthesized UiO-66 Samples

One of the critical issues when dealing with biomedical applications of MOFs is the potential toxicity associated with the structure, due to either the metallic nodes or the organic linkers. To this end, pure and functionalized UiO-66 samples have been evaluated *in vitro* using human retinal pigment epithelium cells (ARPE-19).

**Figure 11:**
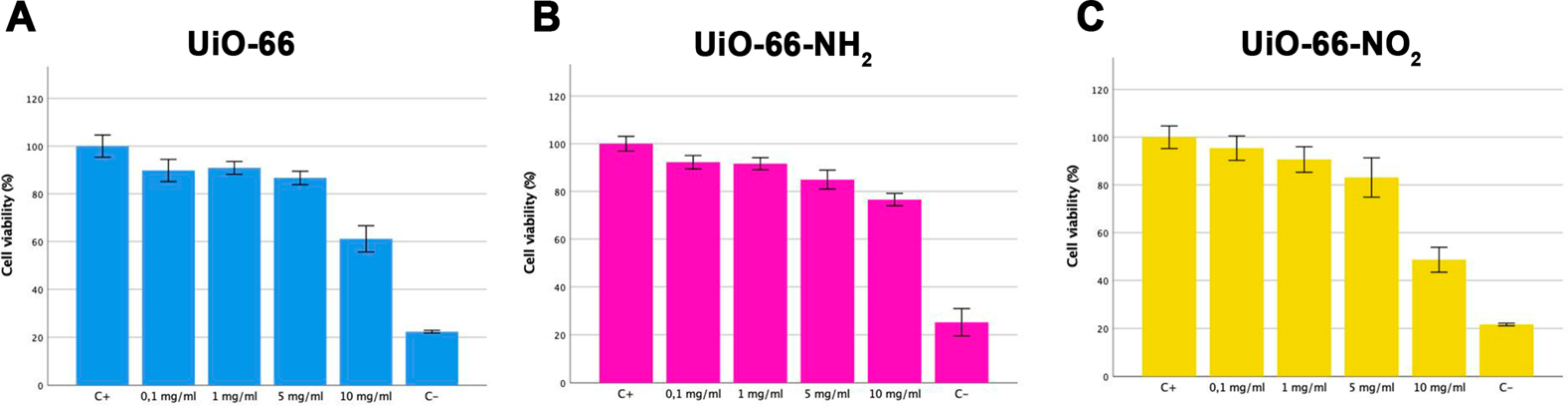
Cell viability assay after 24 h for UiO-66, UiO-66-NH_2_, and UiO-66-NO_2_ using human retinal pigment epithelium cells (ARPE-19) at different MOF concentrations. Positive control cells (C+) were referenced as 100% viability, and negative control cells were treated with ethanol for 15 min (C−). Data represent the mean ± SD.

Compared to the positive (C+) and the negative (C−) controls, Figure 11 shows that all three MOFs exhibit a good biocompatibility at low and medium concentrations (up to 5 mg/mL) with a cell viability above 80%. In other words, Zr-based MOFs exhibit, in general, a high biocompatibility for biomedical applications.^25^ However, viability decreases for high concentrations (10 mg/mL). Under these stringent conditions, UiO-66-NH_2_ preserves a low toxicity (cell viability close to 80%), while UiO-66-NH_2_ and UiO-66-NO_2_ become slightly toxic with a cell viability of 62% and 49%, respectively.

## 4 Conclusions

The functionalization of UiO-66 using −NH_2_ and −NO_2_ groups has been successfully performed using a solvothermal synthesis method under acidic conditions. The synthesized MOFs exhibit reduced textural properties compared to the parent UiO-66 due to the bulkier and heavier functional groups incorporated. Thermogravimetric, XPS, and XRD measurements anticipate the presence of structural defects (missing linkers) in the functionalized samples. The presence of structural defects plays a crucial role in the adsorption/delivery performance for an ocular drug such as brimonidine. Experimental results show that the functionalized MOFs exhibit an improved adsorption performance per volume of cavities (assuming that micropores are the main adsorption sites) with uptake values up to 680 g/cm^3^. Furthermore, these functional groups delay the delivery kinetics up to 25 days vs 10 days in the original, nonfunctionalized, UiO-66-H. Concerning cytotoxicity, *in vitro* experiments using human retinal pigment epithelium cells (ARPE-19) confirm that −NH_2_ functional groups improve the biocompatibility of UiO-66 samples, whereas −NO_2_ groups exhibit a significant cytotoxicity, preferentially at high concentrations. These results emphasize the promoting role of surface functionalities in the design and control of the adsorption and release kinetics for drug molecules and open the gate toward the design of prolonged delivery devices for ocular therapeutics.
